# An expanded taxonomy of hepatitis C virus genotype 6: Characterization of 22 new full-length viral genomes

**DOI:** 10.1016/j.virol.2014.12.025

**Published:** 2015-02

**Authors:** Chunhua Li, Eleanor Barnes, Paul N. Newton, Yongshui Fu, Manivanh Vongsouvath, Paul Klenerman, Hiroaki Okamoto, Kenji Abe, Oliver G. Pybus, Ling Lu

**Affiliations:** a The Viral Oncology Center, Department of Pathology and Laboratory Medicine, University of Kansas Medical Center, Kansas City, KS, USA; b The Peter Medawar Building for Pathogen Research, University of Oxford, South Parks Road and Oxford NIHR Biomedical Research Centre, OX1 3SY, UK; c Lao-Oxford-Mahosot Hospital-Wellcome Trust Research Unit, Microbiology Laboratory, Mahosot Hospital, Vientiane, Lao Democratic People′s Republic; d Centre for Tropical Medicine and Global Health, Churchill Hospital, Nuffield Department of Medicine, University of Oxford, Oxford OX3 7FZ, UK; e Guangzhou Blood Center, Guangzhou 510095, China; f Division of Virology, Department of Infection and Immunity, Jichi Medical University School of Medicine, 3311-1 Yakushiji, Shimotsuke-shi Tochigi 329-0498, Japan; g Department of Pathology, National Institute of Infectious Diseases, Shinjuku-ku, Tokyo 162-8640, Japan; h Department of Zoology, University of Oxford, South Parks Road, OX1 3PS, UK

**Keywords:** HCV, Full-length genome, Genotype 6, Sequence, Laos

## Abstract

We characterized the full-length genomes of 22 hepatitis C virus genotype 6 (HCV-6) isolates: 10 from Vietnam (classified into subtypes 6e, 6h, 6p, 6r, 6s, and 6u), one from China (confirmed as a new subtype 6xd), and 11 from the Lao PDR (representing a new subtype 6xe plus eight novel variants). With these 22 new genomes, HCV-6 now has a diverse and extended taxonomic structure, comprised of 28 assigned subtypes (denoted 6a-6xe) and 27 unassigned lineages, all of which have been represented by full-length genomes. Our phylogenetic analyses also included many partially-sequenced novel variants of HCV-6 from Lao PDR. This revealed that Lao HCV isolates are genetically very diverse and are phylogenetically distributed in multiple lineages within genotype 6. Our results suggest that HCV-6 has been maintained in Laos, a landlocked country, since the common ancestor of genotype 6 and indicates historical dispersal of HCV-6 across Southeast Asia.

## Highlights

• We characterized 22 full-length genomes of HCV-6.
• They confirmed two new subtypes 6xd and 6xe plus eight novel variants.
• We also reanalyzed many partially-sequenced novel HCV-6 variants from Lao PDR.
• They are phylogenetically distributed across the whole of genotype 6.
• They indicate historical dispersal of HCV-6 across Southeast Asia.

## Introduction

Hepatitis C virus (HCV) is a blood-borne pathogen that has a global prevalence of about 3%, affecting approximately 170–220 million people worldwide (WHO, 1999). Infection with HCV results in chronic hepatitis in about 70–85% of the infected individuals, which causes a raised risk of developing cirrhosis, hepatocellular carcinoma, and liver failure (Jahan et al., 2012). The frequency of HCV infection varies considerably among geographic regions, with Egypt displaying the highest prevalence, at 27% (Saeed et al., 1991). In contrast, north-western Europe, North America, and Australia typically exhibit low prevalence of HCV infection, at about 1% (Touzet et al., 2000, Sy and Jamal, 2006). HCV prevalence in Asian countries is variable and in some instances is significantly higher than the global average (WHO, 2012) such that reported in China (3.2%) (Xia et al., 1996), Pakistan (4.95–5.31%) (Khokhar et al., 2004, Muhammad and Jan, 2005), Thailand (3.2–5.6%) (Apichartpiyakul et al., 1999, Songsivilai et al., 1997, Sunanchaikarn et al., 2007), Cambodia (2.3%) (Akkarathamrongsin et al., 2011), and Vietnam (2–9%) (Nakata et al., 1994, Tran et al., 2003). The seroprevalence of anti-HCV among first time blood donors in Vientiane City, Lao PDR (Laos), was detected to be at 1.1% (Jutavijittum et al., 2007), although that study was not a national survey.

Among the seven genotypes of HCV, genotype 6 (HCV-6) exhibits the highest genetic diversity (Salemi and Vandamme, 2002, Wang et al., 2013). Currently, a total of 26 subtypes, denoted 6a-6xc, are formally assigned to HCV-6, and each has at least one full-length genome determined. In addition, 19 lineages have also been completely sequenced, and many novel variants have been detected through the sequencing of partial genomic regions (Smith et al., 2014, Li et al., 2014). Geographically, HCV-6 isolates are typically found in Southeast Asia or among expatriates from this region, which may suggest that this region represents the ancestral and endemic region of HCV-6 (Bernier et al., 1996, Mellor et al., 1996, Noppornpanth et al., 2006, Pham et al., 2011, Pybus et al., 2009, Shinji et al., 2004, Stuyver et al., 1995, Thaikruea et al., 2004, Theamboonlers et al., 2002). In several Southeast Asian countries HCV-6 is highly prevalent. For example, HCV-6 accounts for about 21–49% of HCV infections in Myanmar (Akkarathamrongsin et al., 2011, Lwin et al., 2007, Shinji et al., 2004), 47% in Vietnam (Nguyen et al., 2010, Pham et al., 2009), 56% in Cambodia (Akkarathamrongsin et al., 2011), and a notably high percentage of 95.6% in Laos (Hübschen et al., 2012).

It is not clear why HCV-6 is so dominant in Laos. It has been hypothesized that the globally-prevalent subtypes of 1a, 1b, 2a, and 3a may be rarer in Laos due to the country׳s landlocked environment and historical lack of medical infrastructure, resulting in a lower likelihood of parenteral exposure (Pybus et al., 2009). There have only been two studies of HCV diversity in Laos. The first study investigated samples from 31 patients with anti-HCV antibodies admitted to Mahosot Hospital in Vientiane City. Fifteen patients were found to be HCV RNA-positive and all isolates belonged to HCV-6. Of these, one was classified as subtype 6h, one as subtype 6o, and the remaining 13 isolates represented unclassified variants (Pybus et al., 2009). The second, larger, study investigated samples obtained from 105 first-time blood donors in Vientiane City that were positive for anti-HCV. This resulted in 45 HCV isolates being sequenced in Core-E1 and/or NS5B regions. Among these 45, two represented the globally prevalent subtype 1b, while 43 were classified as HCV-6. Of the 43 HCV-6 isolates, 11 belonged to subtypes 6b, 6h, 6k, 6l, 6n, and 6q, whilst 32 represented novel variants (Hübschen et al., 2011). A common finding from both studies is that Laos contains a very high proportion of novel and unclassified HCV-6 variants that have not been reported in surrounding countries. The high genetic diversity of HCV-6 in Laos suggests the virus has circulated endemically in the country for a very long time.

To better understand the genetic diversity of HCV-6 and to investigate the phylogeny of HCV strains from Laos, we obtained complete genomic sequences representing 11 unclassified HCV-6 variants (Pybus et al., 2009). These new sequences substantially extend the current taxonomic structure of HCV-6 and will form the basis of future studies on the molecular epidemiology and evolution of HCV in Southeast Asia.

## Results

### Genome sequences and organization

Nearly full-length genomes of HCV were determined for 22 HCV-6 isolates (L23, L132, L176, L250, L310, L344, L347, L350, L390, L373, L394, TV280, TV395, TV396, TV407, TV412, TV406, TV462, TV503, TV546, KM98, and VN085), each with 16–20 overlapping fragments (see “Materials and methods” for full sample details). These genomes were 9393–9529 nucleotides (nt) in length, measured from the start of the 5′-UTR through to the variable region or X-tail of the 3′-UTR (Table S1). Each genome contained a single ORF of 9045–9063 nt. The 5′-UTRs were 324–339 nt long, while the 3′-UTRs varied from 1 to 139 nt in length. The sizes of 10 protein encoding regions were the same as those in the H77 reference genome (Table S1) except for the E2 (1089–1107 nt) and NS5A (1053 to 1056 aa) genes, whose lengths varied among the 22 isolates, and for the E1 gene of isolate L350. The latter exhibited a single codon insertion between nucleotide positions corresponding to 1042–1043 in the H77 reference genome.

### Analysis of full-length genomes

**Fig. 1:**
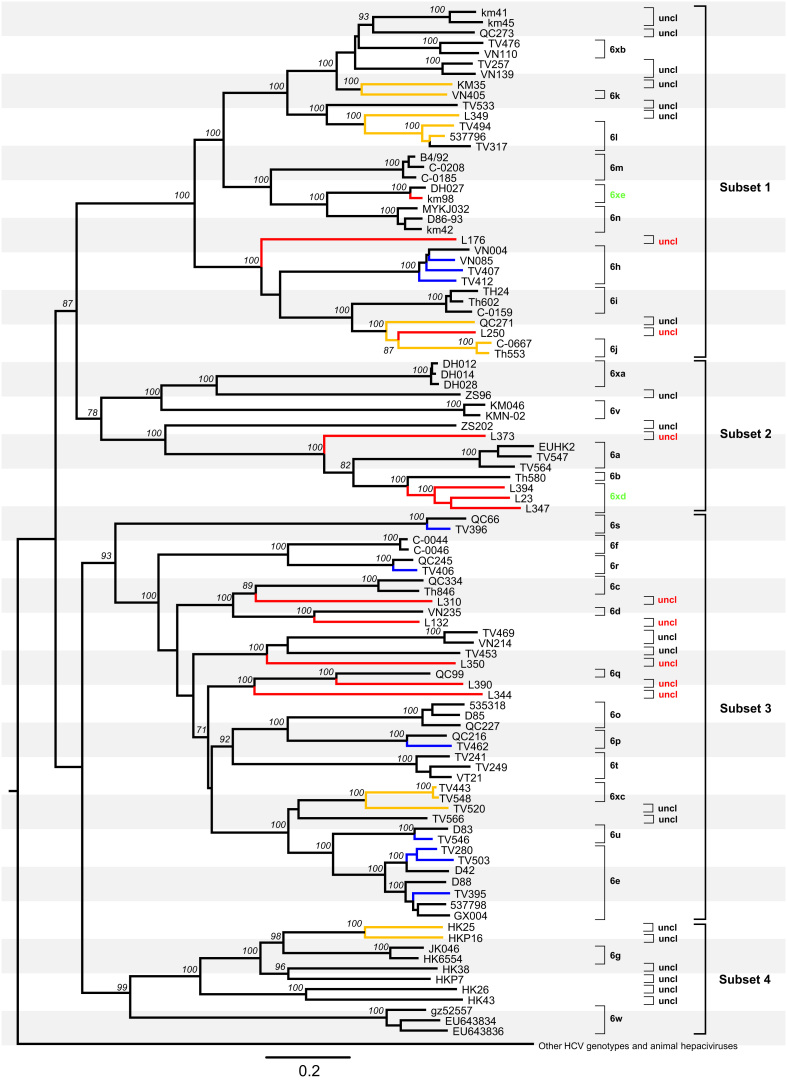
Maximum likelihood tree obtained for 110 full-length HCV genome sequences. The 22 new genomes obtained in this study are in blue (subtypes 6e, 6h, 6p, 6r, 6s, and 6u) or red (new subtypes 6xd, 6xe, and eight unclassified variants). They were analyzed together with 77 HCV-6 full-length references and 11 references from the other six genotypes and 15 sequences of animal hepciviruses. The latter 11 and 15 sequences have been compressed into a single outgroup for clarity. Yellow branches indicate those sequences whose pairwise nucleotide differences fall within a range of 13.5–15%. Subtypes, unclassified lineages, and four subsets discussed in the main text are labelled on the right hand side each. Two new subtypes 6xd and 6xe and eight newly identified unclassified lineages are indicated in green and red, respectively. All others are all indicated in black. Bootstrap supports values >70% are shown in italics at internal nodes, and the scale bar beneath the tree represents 0.10 nucleotide substitutions per site.

A maximum likelihood (ML) tree was obtained based on 99 full-length HCV-6 genome sequences, together with 11 further reference genomes to represent the other six genotypes of HCV (Fig. 1), in addition to 15 sequences of animal hepaciviruses as an outlier group. The latter included three sequences from horses (AB863589, NC024889, and KJ472766), four sequences from bats (KC796074, KC796078, KC796090, and KC796091), and eight sequences from other nonprimate animals (JQ434001–JQ434008). For simplicity, in Fig. 1 all non-HCV-6 genomes and the 15 sequences from animal hepaciviruses have been collapsed into a single branch. Based on phylogenetic analysis and distance-based methods (Simmonds et al., 2005), the 99 HCV-6 sequences were divided into 28 subtypes, 6a-6xe, and 27 unassigned lineages. Each of these exhibited full bootstrap support of 100% in the phylogeny, with two exceptions: the divergence between L310 and subtype 6c (bootstrap score was 89%) and the divergence between L250 and subtype 6j (bootstrap score was 87%). Among the 22 newly-generated sequences from this study, TV280, TV395, and TV503 were classified into subtype 6e, TV407, TV412, and VN085 into 6h, TV462 into 6p, TV406 into 6r, TV396 into 6s, and TV546 into 6u. These 10 genomes, which were all sampled in Vietnam, showed pairwise nucleotide similarities to their nearest references of 87.9–95.0%. Based on the criteria of the current HCV classification system (Simmonds et al., 2005, Smith et al., 2014), these genetic similarities are sufficiently high to classify these 10 isolates into the six subtypes outlined above. With the completion in this study of the genomes for isolates TV280, TV395, and TV503, subtype 6e is now represented by a total of seven full-length sequences. These sequences formed two well-supported clusters in Fig. 1, with TV280, TV503, and D42 belonging to one group and D88, TV395, 537798 and GX004 to the other.

**Fig. 2:**
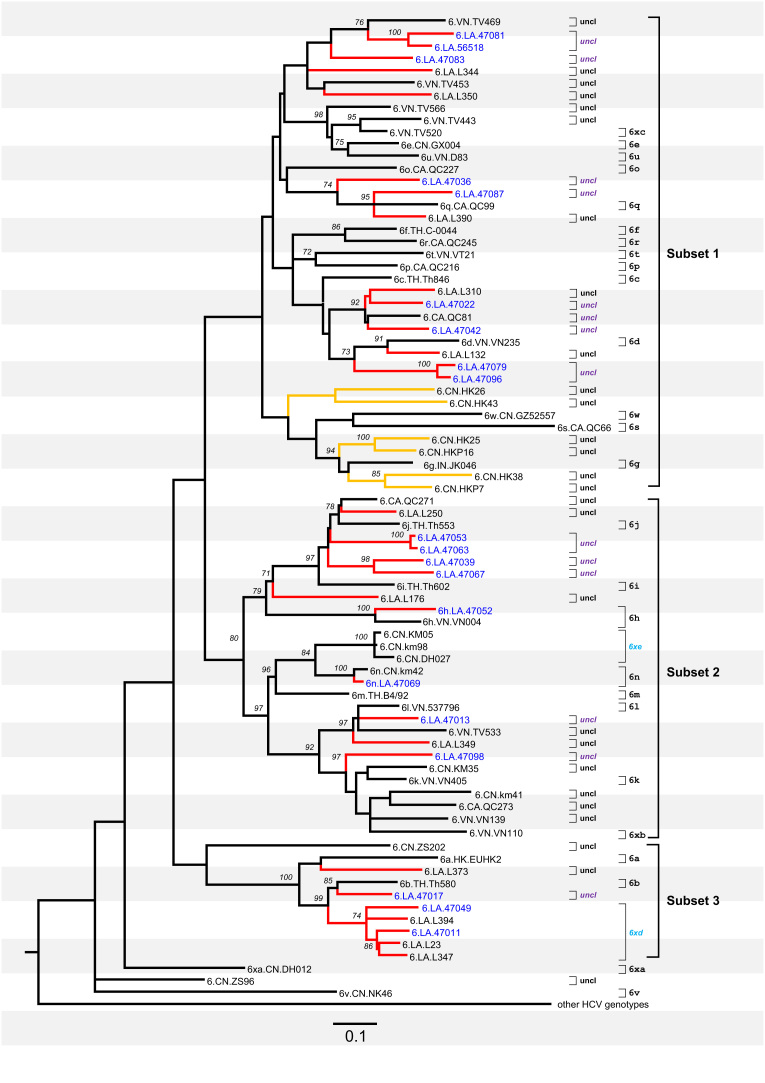
Maximum likelihood tree obtained for 80 core-E1 (Fig. 2) sequences of HCV-6. Red branches represent those sampled in Laos, while black branches represent those sampled in other countries. Subtypes and unclassified lineages are denoted at right hand side. Two new subtypes, 6xd and 6xe, are named in sky blue, while all the unclassified lineages (uncl) are named in black (if completely sequenced) or purple (if partially sequenced). All other annotations are the same as in Fig. 1.

**Fig. 3:**
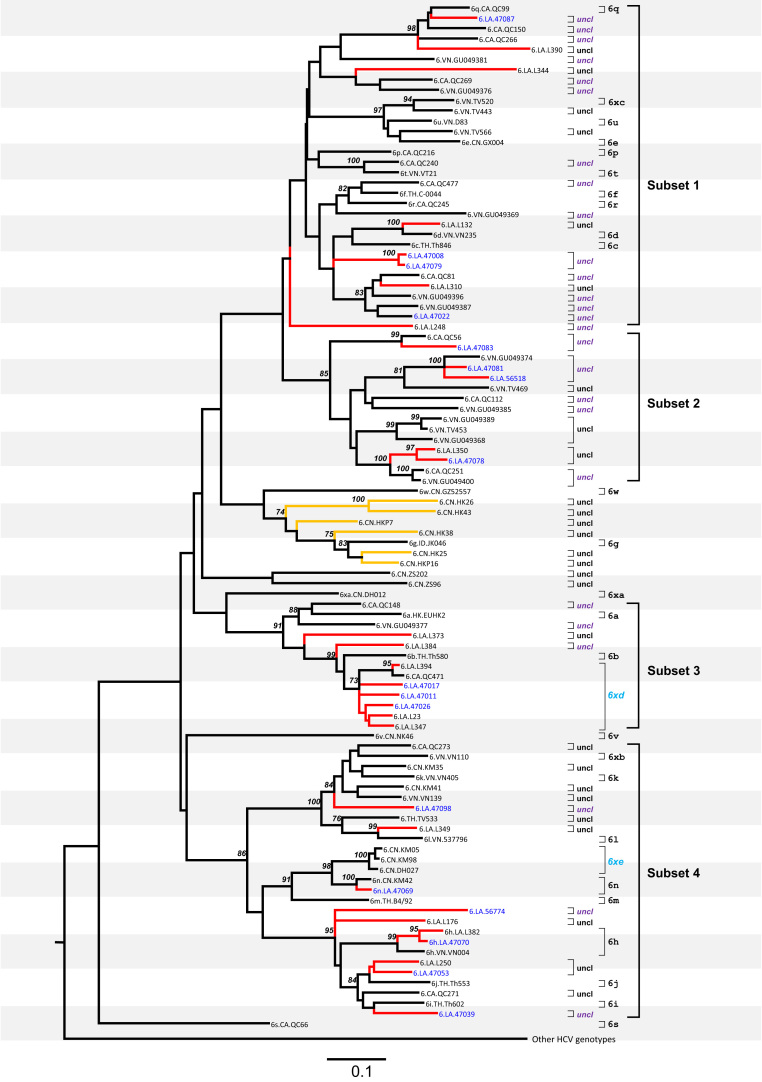
Maximum likelihood tree obtained for 101 NS5B sequences of HCV-6. All the annotations are the same as in Fig. 2.

In contrast, the other 12 genomes obtained in this study could not be classified into any of the currently known subtypes (6a-6xc). Among these, 11 were generated from samples from Laos, and one (KM98) was sampled in China. For convenience of description, we here divided the genetic diversity of HCV-6 into four subsets (Fig. 1). Subset 1 contained three (KM98, L176, and L250) of the 12 unclassified genomes, subset 2 included four (L373, L394, L23, and L347), and subset 3 included five (L310, L132, L350, L390, and L344). In subset 1, KM98 was most closely related to the reference sequence DH027 from China, and the nucleotide similarity between the two was 95%. Further, in the phylogenies obtained for the Core-E1 and NS5B region sequences (Fig. 2, Fig. 3), KM98 and DH027 were joined by another isolate, KM05 (accession numbers KF586079 and KF585672) (Lu et al., 2014). Nucleotide similarities among these three isolates were 95.3–98.4% in the Core-E1 region and 95.3–98.2% in NS5B. These three isolates, which were all sampled in China, represent a new subtype that is denoted 6xd. In subset 2, L23, L347 and L394 formed a cluster that was located close to subtype 6b. Nucleotide similarities among these three genomes were 86.5–88.1% and they differed from the nearest subtype 6b isolate (Th580) in 15.5–15.9%, hence they meet the criteria to confirm a second new subtype, which is denoted 6xe.

The eight sequences from Laos (L132, L176, L250, L310, L344, L350, L390, and L373) each appeared to represent a new subtype but unassigned. Among them, L250 was placed between isolate QC271 and subtype 6j (as represented by isolates C-0667 and Th553) and showed *p*-distances of 14.3%, 14.0%, and 14.1% to the QC271, C-0667 and Th553 sequences, respectively. Excluding isolate L250, the remaining seven Lao sequences all exhibited >15% *p*-distances to their most closely related relatives. In subset 1, L176 was placed close to subtype 6h and differed from 6h by *p*-distances of 21.2–21.8%. In subset 2, L373 was placed near to subtype 6a and differed from 6a by *p*-distances of 21.4–22.1%. In subset 3, L310 was located between subtypes 6c and 6d and showed *p*-distances of 23.5–24.4% from 6c and 6d. L132 was more similar to subtype 6d than to subtype 6c and showed a *p*-distance of 16.8% from 6d. L350 was placed close to TV453 with a *p*-distance of 21%. Both L344 and L390 formed a cluster together with subtype 6q, but they differed from 6q by *p*-distances of 16.6–21.9%.

A range of nucleotide differences, 13–15%, has been recently defined as the genetic distance threshold required to separate isolates of the same subtype from those of different subtypes (Smith et al., 2014). In this study we found HCV-6 genomes whose pairwise nucleotide differences fall within this range, similar to a situation we have recently discussed (Lu et al., 2014). Pairs of isolates affected include HKP16 and HKP25 (*p*-distance=13.6%), L349 and 537796 (*p*-distance=14.3%), QC271 and Th553 (*p*-distance=14.9%), KM35 and VN405 (*p*-distance=14.9%), TV520 and TV443 (*p*-distance=13.6%), and TV520 and TV548 (*p*-distance=13.8%). Similarly, *p*-distances in L250 comparing with QC271, C-0667, and Th553 were 14.3%, 14.1%, and 14.0%, respectively.

The maximized parsimony phylogenies were also reconstructed, which showed topologies almost identical to those shown in ML trees. However, due to the limited space of this paper, we did not present these parsimony phylogenies.

### Similarity plotting

To exclude the possibility of recent viral recombination, pairwise nucleotide similarity values were plotted along HCV genomes using the RDP3 software (Martin et al., 2010). After comparing the 22 full-length genomes that were determined in this study with each other, and with all the reference sequences shown in Fig. 1, no such evidence was detected (data not shown).

### Analysis of partial E1 and NS5B sequences

Recently, a group of HCV Core-E1 and NS5B sequences have been obtained from samples from Laos (Hübschen et al., 2011). Because many of these sequences represent novel HCV-6 variants, here we included them in a separate analysis, which resulted in two ML phylogenies, presented in Fig. 2, Fig. 3. Both phylogenies showed that these subgenomic sequences from Laos were genetically diverse and taxonomically widespread, and many were placed adjacent to Lao sequences whose complete genomes were determined in this study. With a few exceptions, all these partially sequenced Lao isolates exhibited nucleotide differences from their nearest relatives that are above the thresholds calculated in this study for defining the subtypes of HCV-6 in the two regions, Core-E1 and NS5B. Thus they may represent multiple lineages at the subtype level. Because they lack full-length genomes these lineages cannot be confirmed.

Currently, HCV subtypes are no longer defined based on the % of nucleotide differences in subgenomic regions (Smith et al., 2014). However, it may remain useful to calculate *p*-distance thresholds for classifying HCV sequences in the Core-E1 and NS5B regions, because sequences in these two regions are commonly detected and sequenced. Based on this premise, we analyzed all HCV-6 sequences in the core-E1 and NS5B regions in the Los Alamos HCV database and calculated a *p*-distance threshold of 13.6% in the Core-E1 region and of 13.4% in the NS5B region, at which all known HCV-6 subtypes can be differentiated.

The phylogeny in Fig. 2 was obtained based on Core-E1 subgenomic sequences and it can be divided into three subsets for the convenience of description. Based on the above-described *p*-distance threshold of 13.6% for the Core-E1 region, we may tentatively classify these partially sequenced Lao isolates into several lineages. Subset 1 includes nine of these isolates: 47081 and 56518 represent one lineage placed near to TV469; 47083 represents one lineage placed near to L344; 47036 and 47087 represent two lineages placed near to QC99/6q; 47022 and 47042 represent two lineages placed near to L310 and QC081; and 47079 and 47096 represent one lineage placed near to L132.

Subset 2 in Fig. 2 includes eight partially sequenced Lao isolates. Isolates 47053 and 47063 represent one lineage placed near to Th553/6j; 47039 and 47067 represent two lineages placed near to Th602/6i; 47052 is marginally classified as subtype 6h (*p*-distance=14.5%); 47069 is grouped into subtype 6n; and 47013 and 47098 represent two lineages placed near to TV533 and L349.

Subset 3 in Fig. 2 includes three partially sequenced Lao isolates: 47011 represents one lineage placed near to Th580/6b, and 47049 and 47011 form a group with L394, L23, and L347. As described in Fig. 1, the latter three were assigned into the new subtype 6xd and 47049 and 47011 may also belong to this new subtype.

The phylogeny in Fig. 3 was obtained based on the NS5B sub-genomic sequences and it can be divided into four subsets for the convenience of description. Based on the *p*-distance threshold of 13.4% described above for this region, the partially sequenced Lao isolates can be also divided into a number of lineages. Subset 1 includes four partially sequenced Lao isolates: 47008, 47022, 47079, and 47087. Among these, 47087 is classified into subtype 6q, and both 47008 and 47079 represent one lineage placed between Th846/6c and QC81. A group of five sequences (*p*-distances=15.2–18.2%) includes the partially sequenced Lao isolate 47022 and four reference isolates, QC81, L310, GU049396, and GU049387. As a whole, these five sequences have a bootstrap support of 83% and *p*-distances of 9.9–13.7%. As described above for Fig. 2, three isolates in this group (47022, QC81, and L310) clustered with another partially sequenced Lao isolate (47042) and these four exhibited greater *p*-distances of 14.9–17.7%. It is unclear whether all five isolates (i.e. 47022, QC81, L310, GU049396, and GU049387) represent one or several lineages at the subtype level due to the lack of full-length genomes.

Subset 2 in Fig. 3 includes four partially sequenced Lao isolates: 47083 forms a lineage with QC56; 47081 and 56518 form a lineage with GU049374; and 47078 forms a lineage with L350.

Subset 3 in Fig. 3 includes three partially sequenced Lao isolates, 47011, 47017, and 47026, and they all fall into a group with three other Lao sequences (L23, L347, and L394) in addition to QC471. *P*-distances within this group are 2.8–12.7% and it corresponds to the new subtype 6xd proposed in Fig. 1.

Subset 4 in Fig. 3 includes six partially sequenced Lao isolates: 47098 represents a lineage placed between VN139 and TV533; 47069 is classified into subtype 6n; 47070 is classified into subtype 6h; 56774 represents a lineage placed near to L176; 47050 forms a lineage with L250; and 47039 represents a lineage placed near to Th602/6i.

## Discussion

In this study full-length genomes were characterized for 22 HCV-6 isolates. Among them, TV407, TV412, and VN085 were classified into subtype 6h, TV462 into 6p, TV406 into 6r, TV396 into 6s, and TV546 into 6u. These represent the second full-length genomes for each of the five subtypes: 6h, 6p, 6r, 6s and 6u. Evolutionary analysis and viral taxonomy are improved by the inclusion of multiple genetically-distinct genomes per subtype, thereby ensuring a range of genetic diversities from which phylogenetic trees can be obtained (Yuan et al., 2013).

The HCV classification system was updated in 2005 and expanded recently and provides detailed guidelines for defining new subtypes (Simmonds et al., 2005, Smith et al., 2014). According to these guidelines, four isolates from this study enable the confirmation of two new subtypes (KM98 in 6xd and L23, L347, and L394 in 6xe). The existence of subtype 6xe is clear in Fig. 1 and supplemented by four further partially sequenced Lao isolates: 47011, 47017, 47026, 47049, and QC471 in Fig. 2, Fig. 3. The first full-length genome of subtype 6xd, DH027, was reported in Wang et al. (2013) but at that time it represented a solitary unclassified strain. In another study (Lu et al., 2014), we identified a second 6xd isolate (KM05) but that study reported only subgenomic Core-E1 and NS5B sequences. Now that KM98 has been completely sequenced, the new subtype 6xd can be confirmed due to the characterization of three closely related but independent isolates.

Eleven isolates in this study were obtained from samples from Laos. Three were classified into the new subtype 6xe, whilst the remaining eight each represent unclassified HCV-6 lineages for which complete genome sequences are available (because each lacked three closely related isolates) (Smith et al., 2014). With the inclusion of all isolates from previous studies, there are now a total of 27 unclassified HCV-6 lineages with full-length genomes available. Considering that many other divergent strains have been characterized only by subgenomic region sequences, the total number of potential HCV-6 subtypes is undoubtedly large. Such a high genetic diversity is primarily a reflection of the long timescale over which HCV-6 has circulated and diversified in Southeast Asia. Molecular clock analyses estimate that the genotype is at least 1000 years old, and this age is very likely an underestimate of the genotype׳s true age (Pybus et al., 2009). Among the Southeast Asian countries that contain a wide range of different HCV-6 subtypes, Laos has several distinct features; it is a landlocked, mountainous, linguistically diverse country, has a complex history of human migration, and is located in the geographic heart of Southeast Asia (Baird, 2009, Enfield, 2010, Bodner et al., 2011). It is one of the poorest countries in Southeast Asia and few data on HCV prevalence and genotype distribution in Laos are available. Although two previous papers have reported many novel HCV-6 variants from Laos using sub-genomic sequences (Pybus et al., 2009, Hübschen et al., 2011), the sequences obtained were either short or did not exactly match the same subgenomic regions and are therefore not optimal for HCV classification or comparison with other isolates. To better characterize these strains, we determined the full-length HCV-6 genomes of 11 Lao isolates initially reported in Pybus et al. (2009). Although some clusters of Lao isolates can be observed, it is notable that Lao variants are distributed in multiple lineages in the HCV-6 phylogeny, suggesting that HCV-6 has persisted indigenously in Laos for as long as the genotype itself. The high diversity of HCV-6 in Laos is unlikely to have resulted from introductions from other countries during the last century, because HCV-6 subtypes common in Laos, Thailand, Vietnam and elsewhere are interdigitated with each other, indicating historical viral movements among these locations. In other words, although with their earliest origins unknown (in Southeast Asia or other regions?), independent evolution of multiple HCV-6 lineages could have been maintained in the respective geographic regions for many centuries, characteristic of a long-term restricted circulation interspersed with interchanges to a certain extent. However, this shall not necessarily imply that HCV-6 has its earliest origin in Laos, although Laos could be one of the regions to which the earliest HCV-6 strains were introduced. For a better answer to this question, we require further comparison of the HCV-6 genetic diversity in Southeast Asian countries, while in many of these countries such information is still incomplete.

It is worth comparing our findings from Laos with recent results from villages in the central southern mountains of Hainan Island, China, where multiple diverse novel HCV-6 variants have been also discovered (An et al., 2014). The Hainan variants were not spread in multiple lineages in the HCV-6 phylogeny but instead formed a monophyletic cluster with subtypes 6w and 6g that is several hundred years old, but younger than the common ancestor of HCV-6 (An et al., 2014). The villages in Hainan are inhabited by Sino-Tibetan linguistic, Austronesian-descended populations that are thought to have remained from earlier human migrants who moved from Southeast Asia to East Asia during the last ice age, when Hainan Island was connected to the continent (Gray and Jordan, 2000, Li et al., 2008, Peng et al., 2011). It is believed that these populations have lived on Hainan for millennia. The genetic composition of the extant Laos population is complex but is thought to be more similar to Austro-Asiatic and Southern Sino-Tibetan populations than to the Northeast Asian Thai/Tai/Dai or Viet/Kinh populations (Bodner et al., 2011). Further, it is clear that in both Laos and Hainan, endemic HCV-6 has persisted for long periods of time in comparatively remote communities, which has led to a large number of diverse viral strains being generated in geographically-restricted regions. The routes that sustain HCV transmission in such circumstances are unknown and solid evidence is lacking (Pybus et al., 2007). Studies of HCV transmission in remote endemically infected regions should help explain how diverse HCV-6 variants were transmitted and spatially disseminated, and ultimately this information will be useful for improving strategies of HCV prevention and treatment.

Among the pairwise nucleotide differences calculated in this study, some fell in the 13–15% gap observed to differentiate isolates within subtypes from those among subtypes (Smith et al., 2014). This was the case for HKP16 to compare with HKP25, L349 with 537796, KM35 with VN405, TV520 with TV443, and TV520 with TV548, which showed nucleotide differences of 13.6%, 14.3%, 14.9%, 13.6%, and 13.8%, respectively. Similarly, the nucleotide differences in comparing L250 with QC271, C-0667, and Th553 were 14.3%, 14.1%, and 14.0%. Except for VN405 (Tokita et al., 1998), all these full-length genome sequences were characterized in the present and our previous studies, in which we have carefully standardized the experimental conditions to avoid any potential cross contamination, artificial recombination, and errors that may be caused in full-length genome sequencing and assembly (Lu et al., 2007a, Lu et al., 2007b, An et al., 2014, Wang et al., 2013, Li et al., 2014). Hence, we tend to believe that the pairwise nucleotide differences described above would not result from technical problems but would largely reflect the evolutionary history of HCV genotype 6 strains. Theoretically, HCV genetic distances represent a continuum of viral genetic variations resulting from long-term viral evolution, persistently driven by the selective pressure of infected hosts who are also persistently selected by the environments in which they survive. However, due to limited screening or various evolutionary bottlenecks, a large number of these variants may have not been identified or could now be even extinct with only a small portion of them being detected. The above-described 13–15% gap may reflect a temporary threshold which may be narrowed or completely filled as more novel HCV variants are completely sequenced. A part from a few isolates as those described above, nevertheless, this threshold for delimiting subtypes has been shown applicable for the majority of HCV isolates classified to date (Smith et al., 2014).

## Materials and methods

### Subjects and specimens

A total of 22 samples were used in this study. Nine were from HCV-infected individuals in Ho Chi Minh City, Vietnam (Li et al., 2014) and 11 from patients hospitalized at Mahosot Hospital in Vientiane City, Laos (Syhavong et al., 2010, Pybus et al., 2009). Serum VN085 was also from Vietnam but was sampled from a commercial blood donor in 1994 (Tokita et al., 1994). Another serum, KM98, was obtained from a voluntary blood donor in Yunnan province, China (Lu et al., 2014). Partial HCV sequences have been reported from these samples, but not whole genomes.

### PCR amplification and sequencing

From each 100 µl of serum sample, HCV sequences were determined using the approaches we have previously described (Lu et al., 2007a). Briefly, RNA was extracted using the Qiagen Viral RNA extraction system (Qiagen, Valencia, CA) and cDNA was synthesized using the RevertAid First Strand cDNA Synthesis kit (Fermentas Life Science, EU). Based on the resulting cDNA, HCV genomic fragments were amplified to overlap nearly all the full-length genome. An in-house RT-PCR system was used with degenerate HCV-6 primers (Lu et al., 2007a), or in combination with specific primers designed based on the determined sequences. Standard procedures were implemented to avoid potential carryover contamination (Kwok and Higuchi, 1989). Finally the expected amplicons were sequenced (Lu et al., 2007a).

### Phylogenetic analyses and inspection of genome sequences

The 22 genomes obtained were annotated according to the standard nucleotide numbering of the H77 reference genome (Kuiken et al., 2006). To determine phylogenetic relationships, we retrieved 77 full-length HCV-6 sequences for co-analysis, representing 26 assigned subtypes, 6a-6xc (Smith et al., 2014, Li et al., 2014), two new subtypes 6xd and 6xe assigned in this study, and 19 unassigned lineages that we have recently summarized (Li et al., 2014). A full-length HCV sequence dataset was assembled by combining the 22 full-length genomes from this study with an additional 11 sequences representing the other six HCV genotypes (see Supplementary data for detail). Two additional datasets, comprising sub-genomic Core-E1 and NS5B sequences, were also assembled, corresponding to nucleotide positions 869–1289 and 8288–8610 in the H77 genome, respectively. After reducing the number of taxa, these two datasets contained 80 and 101 sequences, respectively (see Supplementary data). These datasets were then aligned using BioEdit (Hall, 1999) followed by a manual adjustment. To exclude potential recent viral recombination events, RDP3 software (Martin et al., 2010) was run with settings as previously described (Lu et al., 2007a).

Using the model test function of MEGA5 software, the best-fitting nucleotide substitution model GTR+I+G was selected according to the corrected Akaike Information Criterion. Based on this model, ML trees were reconstructed for the three HCV-6 datasets using the PhyML software (Guindon and Gascuel, 2003), in which the proportion of invariable sites and gamma distribution shape parameter were estimated directly from the datasets. Base frequencies were adjusted to maximize their likelihoods. Bootstrap analyses of the ML trees were performed using 500 replicates. Pairwise nucleotide similarities/distances were calculated as uncorrected *p*-distances using MEGA 6.0 (Tamura et al., 2013).

### GenBank accession numbers

The nucleotide sequences reported in the present study were deposited in GenBank with the following accession numbers: KM252779-KM252800.

## Financial disclosure

The study described was supported by a grant from the National Institute of Allergy and Infectious Diseases (5 R01 AI080734-03A). The funding agencies had no role in the study design, data collection and analysis, decision to publish, or preparation of the manuscript. The work of Paul N. Newton and Manivanh Vongsouvath are supported by the Wellcome Trust of Great Britain. EB is funded by the Medical Research Council, UK.

## Footnotes

Appendix A Supplementary data associated with this article can be found in the online version at doi:10.1016/j.virol.2014.12.025.
