# Supplementary material for: An expanded taxonomy of hepatitis C virus genotype 6: Characterization of 22 new full-length viral genomes
Source: Virology. 2015 Feb;476:355–63. doi: 10.1016/j.virol.2014.12.025 (PMC4376965; doi:10.1016/j.virol.2014.12.025)
Supplement: Supplementary file 1 — Supplementary data [file mmc1.docx]

| Table S1. The number of nucleotides/amino acids in each genomic region of the 22 HCV-6 isolates * | | | | | | | | | | | | | | | |
| --- | --- | --- | --- | --- | --- | --- | --- | --- | --- | --- | --- | --- | --- | --- | --- |
| Subtype | Isolate | Full | ORF | 5'UTR | Core | E1 | E2 | p7 | NS2 | NS3 | NS4A | NS4B | NS5A | NS5B | 3'UTR |
| 6 | KM98 | 9401 | 9054/3017 | 338 | 573/191 | 576/192 | 1098/366 | 189/63 | 651/217 | 1893/631 | 162/54 | 783/261 | 1353/451 | 1776/591 | 9 |
| 6 | L132 | 9424 | 9051/3016 | 338 | " | " | 1092/364 | " | " | " | " | " | 1356/452 | " | 35 |
| 6 | L176 | 9393 | 9054/3017 | 338 | " | " | 1098/366 | " | " | " | " | " | 1353/451 | " | 1 |
| 6 | L250 | 9418 | 9051/3016 | 338 | " | " | 1095/363 | " | " | " | " | " | 1353/451 | " | 29 |
| 6 | L310 | 9414 | 9054/3017 | 338 | " | " | 1095/363 | " | " | " | " | " | 1356/452 | " | 22 |
| 6 | L344 | 9407 | 9054/3017 | 324 | " | " | 1095/363 | " | " | " | " | " | 1356/452 | " | 29 |
| 6 | L350 | 9422 | 9054/3017 | 338 | " | 579/193 | 1092/364 | " | " | " | " | " | 1356/452 | " | 30 |
| 6 | L390 | 9418 | 9051/3016 | 338 | " | " | 1092/364 | " | " | " | " | " | 1356/452 | " | 29 |
| 6 | L373 | 9428 | 9057/3018 | 341 | " | " | 1101/367 | " | " | " | " | " | 1353/451 | " | 30 |
| 6b | L23 | 9432 | 9060/3019 | 341 | " | " | 1104/368 | " | " | " | " | " | 1353/451 | " | 31 |
| 6b | L347 | 9435 | 9063/3020 | 341 | " | " | 1107/369 | " | " | " | " | " | 1353/451 | " | 31 |
| 6b | L394 | 9434 | 9063/3020 | 341 | " | " | 1107/369 | " | " | " | " | " | 1353/451 | " | 30 |
| 6e | TV280 | 9459 | 9057/3018 | 338 | " | " | 1101/367 | " | " | " | " | " | 1353/451 | " | 64 |
| 6e | TV395 | 9440 | 9060/3019 | 338 | " | " | 1101/367 | " | " | " | " | " | 1356/452 | " | 33 |
| 6e | TV503 | 9420 | 9057/3018 | 338 | " | " | 1101/367 | " | " | " | " | " | 1353/451 | " | 25 |
| 6h | TV407 | 9529 | 9051/3016 | 339 | " | " | 1095/363 | " | " | " | " | " | 1353/451 | " | 139 |
| 6h | TV412 | 9426 | 9045/3014 | 324 | " | " | 1089/361 | " | " | " | " | " | 1353/451 | " | 57 |
| 6h | VN085 | 9409 | 9051/3016 | 337 | " | " | 1095/363 | " | " | " | " | " | 1353/451 | " | 21 |
| 6p | TV462 | 9424 | 9051/3016 | 339 | " | " | 1092/364 | " | " | " | " | " | 1356/452 | " | 34 |
| 6r | TV406 | 9457 | 9051/3016 | 338 | " | " | 1092/364 | " | " | " | " | " | 1356/452 | " | 68 |
| 6s | TV396 | 9418 | 9051/3016 | 338 | " | " | 1095/363 | " | " | " | " | " | 1353/451 | " | 29 |
| 6u | TV546 | 9456 | 9057/3018 | 338 | " | " | 1098/366 | " | " | " | " | " | 1356/452 | " | 61 |
| * The H77 genome (GenBank accession no. AF009606), HCV genotype 1a, is referenced. Bold entries indicate the regions are variable in length. | | | | | | | | | | | | | | | |
